# Genomic analysis reveals genetic diversity and selection signatures of the Yantai Black pig during domestication and breeding

**DOI:** 10.3389/fgene.2025.1730668

**Published:** 2025-11-25

**Authors:** Ming Qin, Cai Ma, Mingzhi Liang, Yuxin Zhang, Zengguang Wang, Jingyu Wang, Guodong Li, Yufen Sha, Peng Jin, Lingling Ju, Xueping Liu, Yongqing Zeng, Ruili Li

**Affiliations:** 1 Institute of Animal Science and Veterinary Medicine, Yantai Academy of Agricultural Sciences, Yantai, China; 2 Shandong Provincial Key Laboratory of Animal Biotechnology and Disease Control and Prevention, College of Animal Science and Technology, Shandong Agricultural University, Taian, China; 3 Department of Medical Genetics and Cell Biology, Binzhou Medical University, Yantai, China; 4 Yantai Agricultural Bureau, Yantai, China; 5 Laizhou Agricultural Bureau, Yantai, China

**Keywords:** Yantai Black pig, population genetics, selection regions, candidate genes, whole-genome resequencing

## Abstract

**Objective:**

Yantai Black pig (YT), as a native population of the eastern China’s Jiaodong Peninsula of Shandong province, characterized by coarse feeding tolerance, strong disease resistance, early sexual maturity, high litter size, and superior meat quality. However, the genetic characteristics and variations underlying its crucial economic traits remain poorly understood.

**Methods:**

In this study, we resequenced the whole genome of 17 YT individuals from distinctly different lineages breeding in three conservation farms to detect single nucleotide polymorphism (SNP) density, pairwise fixation index (*F*_ST_), nucleotide diversity (π), runs of homozygosity (ROHs).

**Results:**

Our findings revealed that YT has higher genomic diversity compared to Chinese partial indigenous pig populations and Western commercial pig populations, but lower diversity than Asian wild boars (AWB). Based on *F*_ST_ and values (top 1%), we identified 321 selected regions, encompassing 156 genes, between YT and AWB. Functional annotation analysis suggested that these genes are potentially responsible for growth, reproduction, and immune responses. The *RBFOX3* and *WDR27* genes were confirmed to be strong positively selected in YT’s breeding. Combining the results of selection sweeps and ROH islands of YT, three overlapping regions were detected. Furthermore, we found that the quantitative trait loci (QTLs) with the most overlapping regions were related to teat number, body weight, and mean corpuscular hemoglobin concentration.

**Conclusion:**

We characterized the genomic features and population structure and identified selection signals in genomic regions linked to important germplasm characteristics of YT. The insights gained from this study provide valuable references and a solid foundation for the preservation, breeding, and utilization of YT and its valuable genetic resources.

## Introduction

**FIGURE 1:**
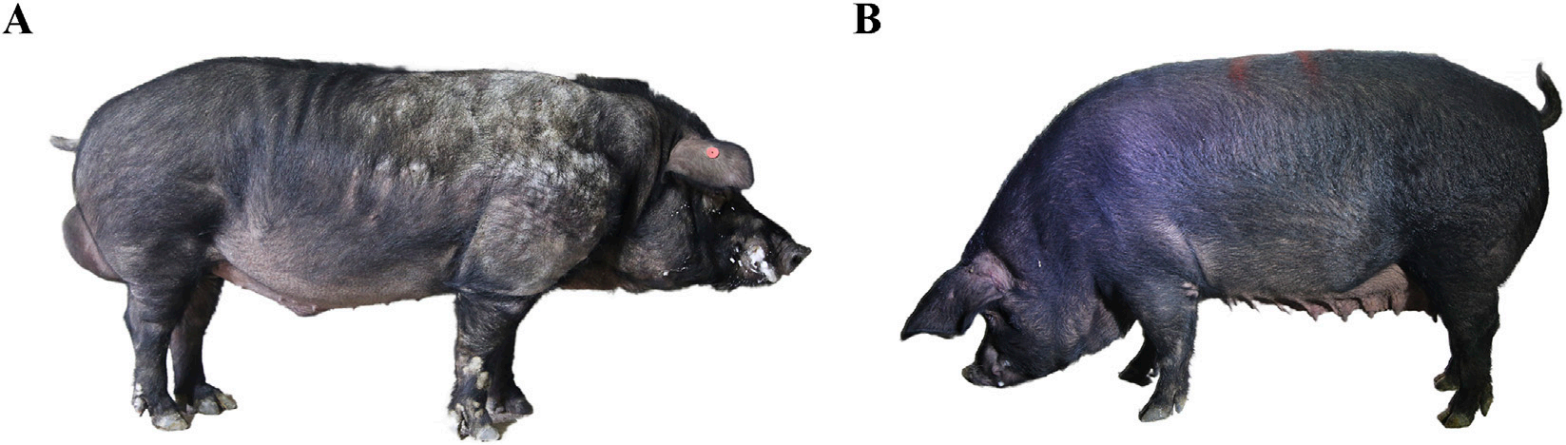
Appearance of Yantai Black pig. **(A)** Boar. **(B)** Sow.

The domestic pig (*Sus scrofa*), a widely raised livestock species, was domesticated approximately 9,000 years ago (Chen et al., 2007). This extensive history has enabled pigs to adapt to a variety of environments, resulting the emergence of diverse populations. Over time, both natural and artificial selection have favored certain economic traits, while distinct historical and geographical demands for meat production have led to significant phenotypic differences between Asian and European pig populations. Notably, Asian populations tend to exhibit higher prolificacy, increased fat accumulation, and slower growth rates compared to European counterparts (Cho et al., 2019; Wang et al., 2021). In particular, genetic resources from Chinese indigenous pigs have been extensively utilized to enhance various commercial populations. The Yantai Black pig (Figure 1), a significant indigenous population from Shandong province, China, is renowned for its coarse-feeding tolerance, strong adaptability, high fecundity, and superior meat quality. This population exhibits significant advantages in terms of meat antioxidant properties when compared to both other Chinese indigenous populations and introduced populations (Liu, 2018; Min et al., 2018). There are currently three Yantai Black pig conservation breeding farms, representing six different families, located in Weihai and Laizhou City. More than 50 breeding boars and more than 1000 sows make up the core herd at these locations. However, although the Yantai Black pig is acknowledged as an indigenous population, data regarding its genetic diversity are relatively sparse. Therefore, further research is essential to investigate and understand the genetic traits and diversity within this population.

The swift progress in sequencing technology have established genome resequencing as an indispensable tool in genetic research. Multiple investigations have utilized genome resequencing to examine the genetic elements that impact economically relevant traits and production performance in pigs (Zhang et al., 2024a). Key areas of investigation include reproductive capabilities (Li et al., 2020), meat quality (Wu et al., 2020), growth rates (Zhou et al., 2023), and immune responses (Qi et al., 2024). This methodology is crucial for the advancing the local pig industry, as it facilitates the discovery of genetic potential within pig genomes. Identifying beneficial genes is essential for developing effective breeding strategies that improve the adaptability and productivity of pigs. However, the processes of domestication and commercialization can significantly impact associated genomes, primarily reducing genetic diversity and changes in population structure. Gaining insights into the genetic impacts of domestication and commercialization is essential for preserving genetic variability and addressing the potential challenges posed by intensive breeding and management practices in pig populations.

Recent research has pinpointed particular genomic regions linked to the previously mentioned traits, thereby aiding in the detection of beneficial selection signals (Du et al., 2024). Nevertheless, investigations into the genomic information of the Yantai Black pig are still relatively restricted. Thus, this study had the following aims: (1) to detect the differences in genetic structure between YT and various pig populations using whole-genome resequencing; (2) to identify the occurrence and distribution of ROHs in YT and AWB; (3) to calculate and compare the genomic inbreeding coefficients (*F*_ROH_) between YT and AWB using ROHs; (4) to identify and compare potential selective regions associated with economically important traits in YT. The findings will serve as a foundation for further research on the genetic underpinnings of key economic traits at the genome-wide level and offer insights to facilitate the efficient implementation of pig breeding strategies.

## Materials and methods

### Sample collection and whole-genome resequencing

We selected 17 Yantai Black boars for the trial from two genetic resource preserving farms and one expansion farm in Shandong Province, ensuring that they conformed to the population characteristics and features (DB37/T 4573. 11–2024), with each pig possessing complete pedigree records from the conservation farm’s pedigree registry. To achieve a comprehensive representation of the YT population lineage, we collected at least two boars from each lineage. Genomic DNA was isolated from ear tissues utilizing the cetyltrimethylammonium bromide (CTAB) method (Sambrook et al., 1983). The genomic DNA purity was evaluated via a NanoDrop 2000 spectrophotometer (Thermo Fisher Scientific, Waltham, MA, USA), ensuring an optimal OD260/OD280 absorbance ratio within the range of 1.8–2.0. Additionally, DNA concentration was measured using a Qubit® 4.0 fluorometer. Paired-end libraries with a 500 bp insert were generated using the Paired-End DNA Sample Prep kit (Illumina Inc, San Diego, CA, USA), and sequencing was carried out on the NovaSeq 6000 (Illumina Inc, San Diego, CA, USA) NGS platform. Furthermore, we obtained genomic data from 21 individuals across seven populations from the Sequence Read Archive (SRA) database, including Yorkshire pig (Y, n = 3), Landrace pig (L, n = 3), Duroc pig (D, n = 3), Erhualian pig (EHL, n = 3), Min pig (MIN, n = 3), Laiwu pig (LW, n = 3) and Asian wild boar (AWB, n = 3). In total, genomic data from 38 individuals were subsequently analyzed (Table 1).

**TABLE 1:** Overall details of eight pig populations.

Population	Samples	Origin	Average sequencing coverage
YT	17	Yantai city	11.68×
EHL	3	SRR949637; SRR949639; SRR949640	14.22×
MIN	3	SRR949729; SRR949731; SRR949733	13.61×
LW	3	SRR949753; SRR949755; SRR949761	13.19×
Y	3	SRR1581141; SRR1581140; SRR1581138	14.05×
L	3	SRR1581026; SRR1581043; SRR1581044	13.60×
D	3	SRR1577860; SRR1577862; SRR1577866	17.36×
AWB	3	SRR949643; SRR949645; SRR949648	13.14×

YT, Yantai Black pig; EHL, erhualian pig; MIN, min pig; LW, laiwu pig; Y, yorkshire pig; L, landrace pig; D, duroc pig; AWB, asian wild boar.

### Quality control and comparative analysis of sequencing data

Raw reads would be processed to get high quality clean reads according to four stringent filtering standards: 1) removing reads with ≥10% unidentified nucleotides (N); 2) removing reads with > 50% bases having phred quality scores of ≤20; 3) removing reads aligned to the barcode adapter. For SNP and Indel identification, the Burrows-Wheeler Aligner (BWA-0.7.15) was used to align clean reads from each sample against the *S. scrofa* 11.1 reference genome with settings ‘mean 4 -k 32 -M’, -k is the minimum seed length, and -M is an option used to mark shorter split alignment hits as secondary alignments (Li and Durbin, 2009). Variant calling for all samples was conducted utilizing GATK’s Unified Genotyper (v3.5-0-g36282e4). Subsequently, SNPs and Indels were filtered with GATK’s Variant Filtration with proper standards (-Window 4, -filter “QD < 2.0 || FS > 60.0 || MQ < 40.0”, -G_filter “GQ < 20”) and those exhibiting segregation distortion or sequencing errors were discarded. The physical positions of each SNP were established using ANNOVAR (2017 June 01) (Wang et al., 2010) for alignment and annotation of SNPs and InDels. Structural variants, including translocations, inversions, and insertion, were identified using software BreakDancer (Max1.1.2) (Chen et al., 2009). Copy number variants (CNVs) were categorized using CNVnator (0.3.2) (Abyzov et al., 2011).

### Genetic diversity analysis and linkage disequilibrium

A phylogenetic tree was constructed using the neighbor-joining method implemented in PHYLIP (3.69) (Plotree and Plotgram, 1989), with confidence intervals estimated through 1000 bootstrap iterations. Initial classification of population subdivision patterns was conducted using principal component analysis (PCA) with GCTA (Yang et al., 2011). To further evaluate population structure, the admixture model-based software Admixture (1.3.0) (Alexander et al., 2009) was employed to estimate the Q matrix. The tested values of K ranged from two to 9, with the optimal K determined by the lowest cross-validation error. The kinship matrix was derived using the SPAGeDi (1.5) (Hardy and Vekemans, 2002). To assess the linkage disequilibrium (LD) pattern, the squared allele frequency correlation (r^2^) was computed using Haploview (4.2) (Barrett et al., 2005), with the following parameters: maxdistance 1000 -dprime -minGeno 0.6 -minMAF 0.05 -hwcutoff 0.

### Detection of genome-wide selection signatures

To eliminate indels from the markers, Vcftools (0.1.14) (Danecek et al., 2011) was used, followed by filtering SNP sites using PLINK (1.90b3.36 64-bit) (Purcell et al., 2007) option--indep-pairwise 50 10 0.1. ROHs were detected using the following parameters: -homozyg-density 100 --homozyg-gap 1000 --homozyg-kb 100 --homozyg-snp 50 --homozyg-window-het 1 --homozyg-window-missing 5 --homozyg-window-snp 50 --homozyg-window-threshold 0.05. Significant overlapping or adjacent windows were merged into larger genomic regions. The distribution of ROH fragments indicates variations in both length and quantity among different populations, reflecting shifts in ROH. The genome inbreeding coefficient was calculated using ROH to assess the degree of inbreeding within populations. The calculation is as follows: *F*_ROH_ = $\sum \frac{LROH}{LAUTO}$ . Utilizing the population as a reference, the ROH ratio was calculated for each SNP site within ROH regions. The threshold for high-frequency SNPs was set as the top 1% ROH ratio, and ROH islands were identified based on the distribution of SNP sites exceeding this threshold within the genome.

The selective sweep regions were identified based on the top 1% (*F*_ST_ ≥ 0.49 and π ratio ≥3.25) and 5% (*F*_ST_ ≥ 0.39 and π ratio ≥2.12) of *F*_ST_ and π ratio (π_AWB_/π_YT_) (Li et al., 2013). Tajima’s D value (Tajima, 1989) was used to assess whether the candidate selective scanning regions contained excessive singleton polymorphisms. A sliding window approach of 100 kb, with a step size of 10 kb, was used to compute these parameters using the PopGenome package (Pfeifer et al., 2014). All associated graphs were generated using R scripts. Candidate genes within the sweep regions were retrieved for subsequent analysis.

### Annotation of candidate genes and identification of QTL that overlap with selection signatures and ROH islands

To enhance the understanding of the functions of the identified genes, gene ontology (GO) and Kyoto Encyclopedia of Genes and Genomes (KEGG) pathways enrichment analyses were conducted using the KOBAS web-based platform (Bu et al., 2021). Additionally, the pig QTL database (https://www.animalgenome.org/cgi-bin/QTLdb/SS/index) was used to identify overlapping regions between selection sweeps and ROH islands associated with trait-related selective signatures.

## Results

### Sequencing variants of Yantai Black pig

**FIGURE 2:**
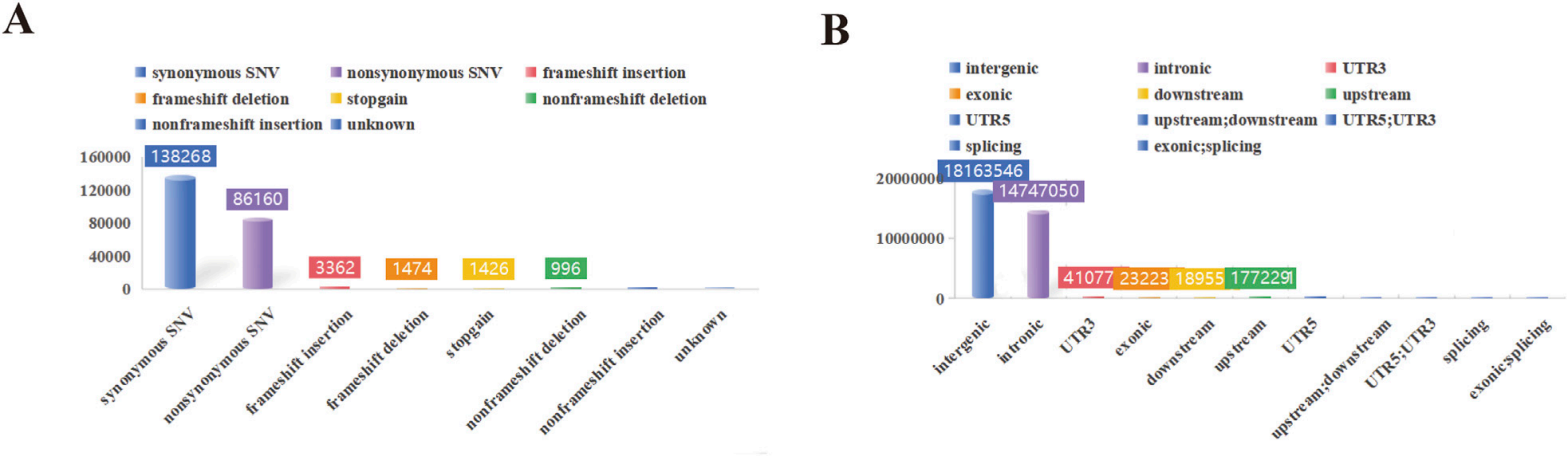
SNP detection and distribution. **(A)** The functional annotation statistical map of genetic variation. **(B)** The statistical map of genetic variation locations.

In this study, a total of 1,293.27 Gb of raw data was collected for 38 swine genomes. The average sequencing depth was 11.68X, with variations ranging from 11.10X to 12.45X. Summary statistics for the resequencing data are presented in Supplementary Table S1. Among all samples, a total of 30,436,852 SNPs were detected in YT. Furthermore, YT exhibited 3,623,390 indels among the 38 samples. Notably, YT exhibited a significantly higher number of transition SNPs compared to transversion SNPs, with a transition/transversion ratio of 2.51 (Supplementary Table S2). The SNP annotation results revealed that these variations primarily occurred in intergenic regions, with the least prevalence in exonic; splicing (Figure 2).

### Analysis of genetic diversity and linkage disequilibrium

**FIGURE 3:**
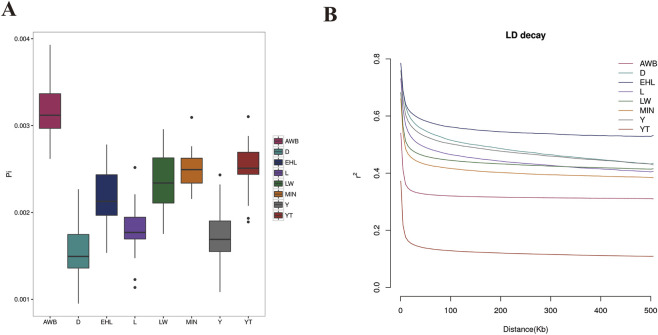
The analysis of nucleotide diversity and linkage disequilibrium decay among eight pig populations. YT, Yantai Black pig; EHL, Erhualian pig; MIN, Min pig; LW, Laiwu pig; Y, Yorkshire pig; L, Landrace pig; D, Duroc pig; AWB, Asian wild boar. **(A)** Box-plot of the nucleotide diversity (π) for each population. **(B)** Genome-wide mean decay of linkage disequilibrium for each population.

To assess the extent of variation in nucleotide sequences among individuals within each pig population, we calculated nucleotide diversity. The findings indicated that nucleotide diversity was highest in AWB (0.003163), followed by YT (0.002501) (Figure 3A). Conversely, lower LD decay was observed in YT than that in AWB (Figure 3B).

### Population structure and admixture analysis

**FIGURE 4:**
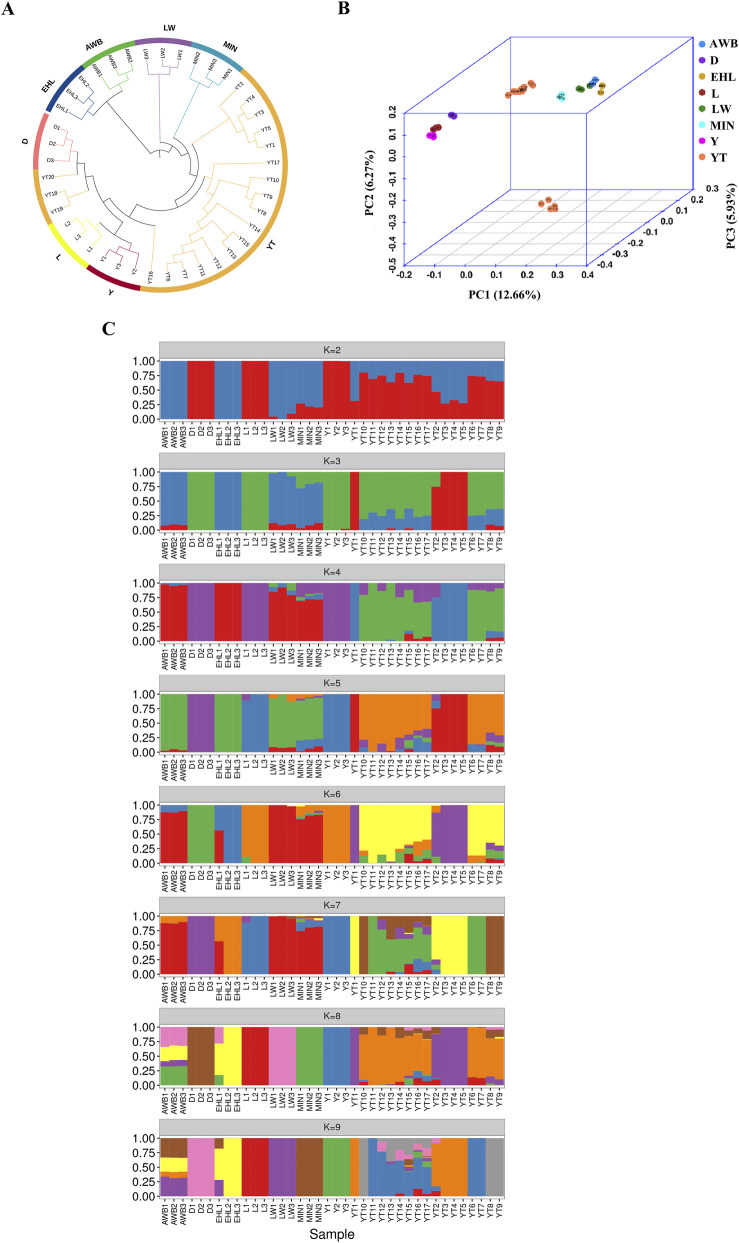
The analysis of population structure among eight pig populations. YT, Yantai Black pig; EHL, Erhualian pig; MIN, Min pig; LW, Laiwu pig; Y, Yorkshire pig; L, Landrace pig; D, Duroc pig; AWB, Asian wild boar. **(A)** The neighbor-joining tree of relationships for each population. **(B)** The principal component analysis for each population. **(C)** The genetic structure for each population using ADMIXTURE with K = 2 and K = 8.

To investigate the genetic connections between the YT and various pig populations, we analyzed population structure using autosomal SNPs. The neighbor-joining (NJ) tree initially showed that the YT was positioned between Chinese indigenous pig populations (MIN, LW, and EHL) and Western commercial populations (D, L, and Y), with AWB clustering closely with EHL and LW (Figure 4A). PCA supported these findings, yielding similar results (Figure 4B). The first principal component explained 12.66% of the total genomic variation, while the second principal component accounted for 6.27%. ADMIXTURE analysis revealed the ancestral components of all individuals evaluated. We analyzed K values ranging from two to nine to observe admixture proportions. At K = 2, the coefficient of variation (CV) was minimal, indicating the most appropriate grouping. Distinctions emerged between the Chinese indigenous pig populations and Western commercial pig populations, with the YT showing significant ancestry from Western populations and a genetic influx from AWB. By K = 8, the four Chinese indigenous pig populations were distinctly separated, but some YT individuals exhibited mixed characteristics (Figure 4C).

### Genomic distribution of ROH

In this study, a total of 24,392 ROHs were detected within the YT and AWB populations, with counts of 21,833 and 2,559, respectively. On average, each individual pig from the YT group had 1,878.5 ROHs, ranging from 1,616 to 2,170. We summarized ROHs categorized by length, with average lengths ranging from 213.04 kb to 509.57 kb (Supplementary Table S3). The overall count of ROHs in the YT population primarily consisted of shorter segments (0–1 Mb), which constituted 95.14% of all ROH fragments, compared to segments exceeding 1 Mb (4.86%). There were 11,026 common ROHs in YT and 2,540 in AWB, respectively (Table 2). Furthermore, the predominant common ROH length for both YT and AWB was within the 0.1–0.5 Mb range. At the chromosomal level, SSC one had the highest count of common ROHs (n = 1,682) in YT, while SSC 10 accounted for the fewest (n = 276) (Supplementary Figure S1). The greatest proportion of common ROHs exceeding 1 Mb was in SSC 16 (25.03%), while the least was in SSC 5 (8.20%).

**TABLE 2:** The distribution of common ROH among two pig populations.

Population	ROH length (Mb)	ROH number	Percentage (%)	Mean length (Mb) ± SD
YT	0.1–0.5	8437	76.52	0.25 ± 0.10
	0.5–1	1825	16.55	0.69 ± 0.14
	>1	764	6.93	1.57 ± 0.60
Total		11,026	100	
AWB	0.1–0.5	2268	89.29	0.22 ± 0.09
	0.5–1	183	7.21	0.67 ± 0.13
	>1	89	3.50	1.87 ± 0.89
Total		2540	100	

YT, Yantai Black pig; AWB, asian wild boar; ROH, runs of homozygosity.

**FIGURE 5:**
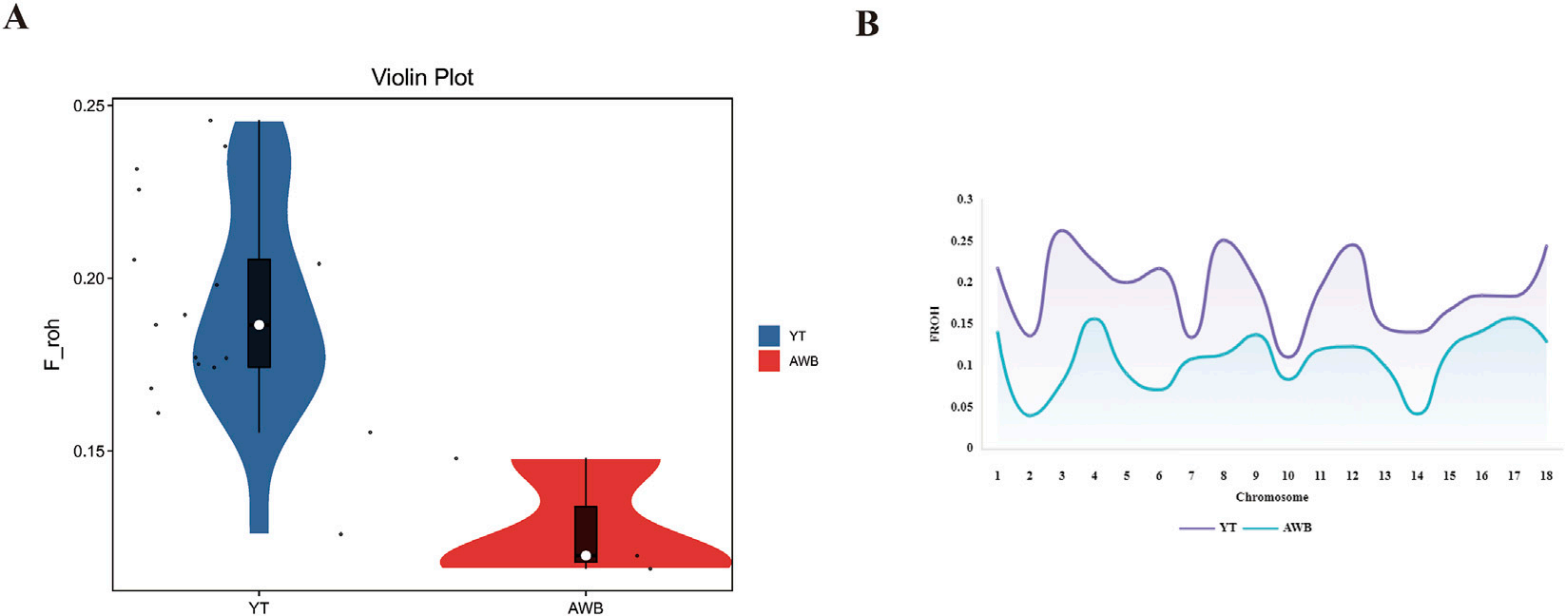
Individual *F*_ROH_ and Chromosome *F*_ROH_ statistics of YT and AWB. YT, Yantai Black pig; AWB, Asian wild boar. **(A)** The *F*_ROH_ of each individual. **(B)** Distribution of *F*_ROH_ based on ROH for each chromosome.

The distribution of individual-level *F*_ROH_ (Supplementary Table S4) and *F*_ROH_ across various chromosomes is shown in Figure 5A. The average *F*_ROH_ values for YT and AWB were 0.2 and 0.12 (Figure 5B), respectively, indicating variations among chromosomes. Notably, SSC three had elevated *F*_ROH_ values. Furthermore, Table 3 showed *F*_ROH_ values categorized by different length levels.

**TABLE 3:** Descriptive statistics for *F*_ROH_ among two pig populations.

Population	*F*_ROH_ (0.1–0.5 Mb) ± SD	*F*_ROH_ (0.5–1 Mb) ± SD	*F*_ROH_ (>1 Mb) ± SD	*F*_ROH_ ± SD
YT	0.11 ± 0.01	0.05 ± 0.01	0.04 ± 0.01	0.20 ± 0.03
AWB	0.07 ± 0.01	0.02 ± 0.00	0.02 ± 0.01	0.12 ± 0.01

YT, Yantai Black pig; AWB, asian wild boar.

### Genome-wide scanning for selection signatures

**FIGURE 6:**
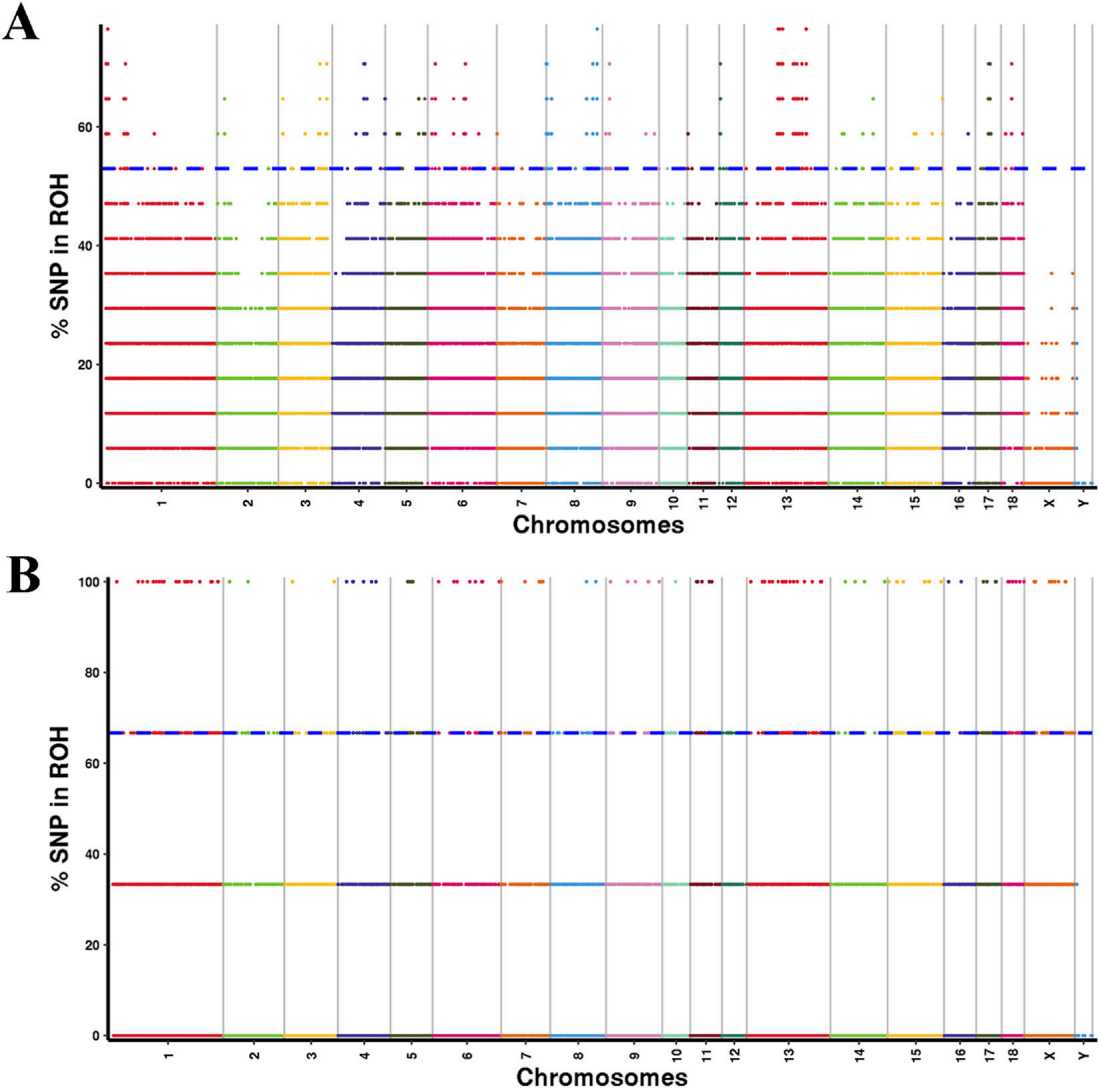
Manhattan plot of the frequency (%) of each SNP in the runs of homozygosity (ROH) for YT **(A)** and AWB **(B)**.

To pinpoint the genomic regions most strongly associated with ROH across all individuals, SNPs within the top 1% frequency present in at least 52.94% of YT samples and 66.67% of AWB samples were selected as potential candidate SNPs (Figure 6). In the YT population, 275 ROH islands were identified, characterized by uneven distribution across chromosomes and lengths ranging from 0 to 1238.82 kb (Supplementary Table S5). The largest ROH island was located on chromosome SSC 1, comprising 621 adjacent SNPs and containing eight candidate genes. Among the 662 candidate genes identified within the ROH islands of YT, 250 KEGG pathways were notably enriched, with significant enrichment in pathways related to Glycosaminoglycan degradation and Serotonergic synapses (Supplementary Figure S2). Analysis of overlapping ROH island regions in the genomes of the two pig populations revealed significant overlaps occurred on chromosomes SSC 4, 5, and 6. Additionally, overlapping ROH island regions were detected on SSC 9 (133.4 Mb–133.59 Mb, GRB10) and SSC 15 (9.99 Mb–10.26 Mb, LRP1B in both populations (Supplementary Table S6).

**FIGURE 7:**
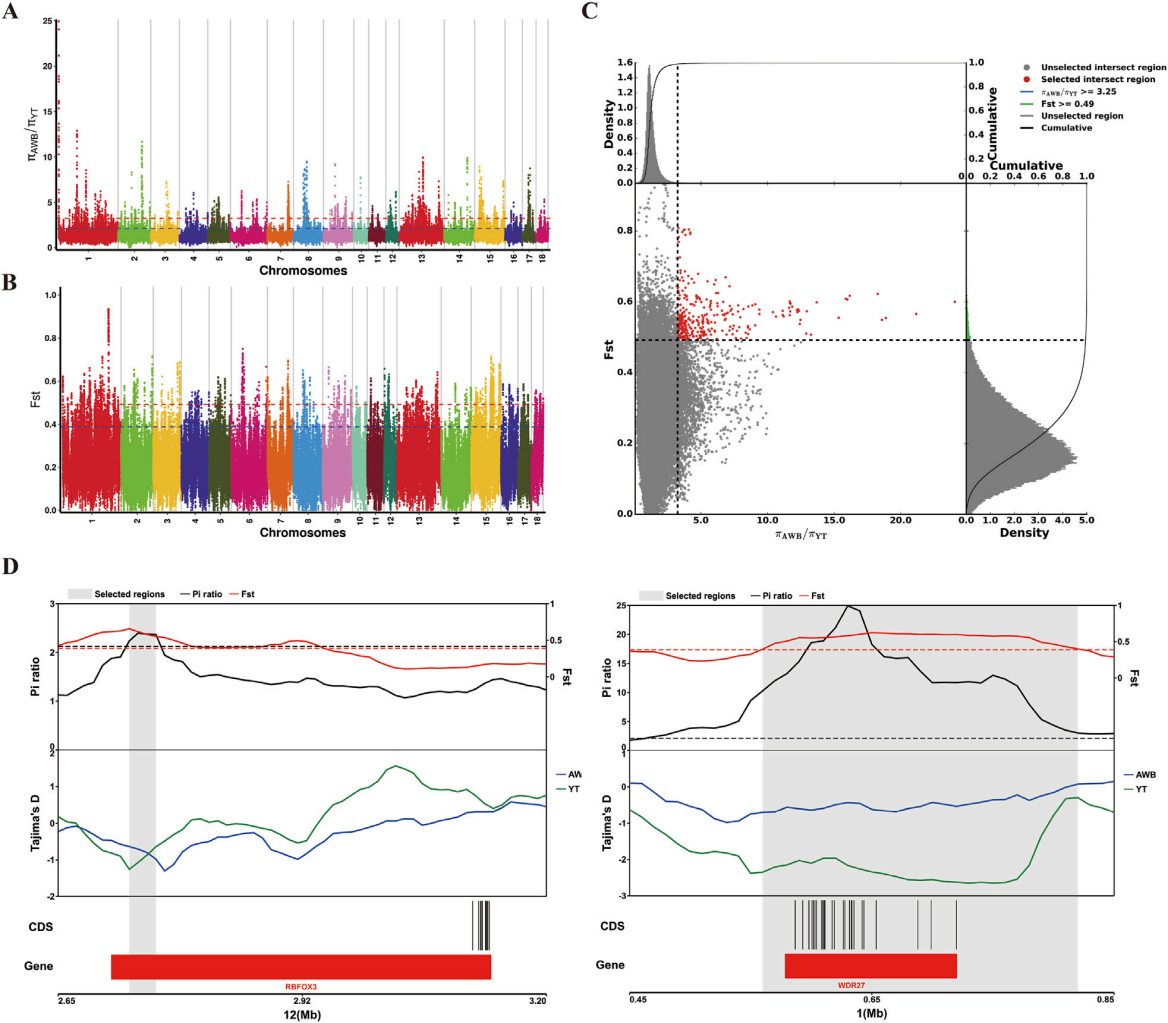
Analysis of the signatures of positive selection in the genome YT compared to AWB. YT, Yantai Black pig; AWB, Asian wild boar. **(A)** Manhattan plot of selective sweeps using π ratio in YT vs. AWB. **(B)** Manhattan plot of selective sweeps using *F*_ST_ in YT vs. AWB. **(C)** Distribution of π ratio (AWB/YT) and *F*_ST_ values, which are calculated in 100 kb windows sliding in 10 kb steps, Data points located to the right of the vertical dashed lines respectively (corresponding to the 1% right tail of the empirical π ratio distribution where the π ratio is 3.25) and above the horizontal dashed line (the 1% right tail of the empirical *F*_ST_ distribution, where *F*_ST_ is 0.49) were identified as selected regions for YT (red points). **(D)** Example of genes with strong selective sweep signal in YT. π ratio and *F*_ST_ values are plotted using a 10 kb sliding window.

**FIGURE 8:**
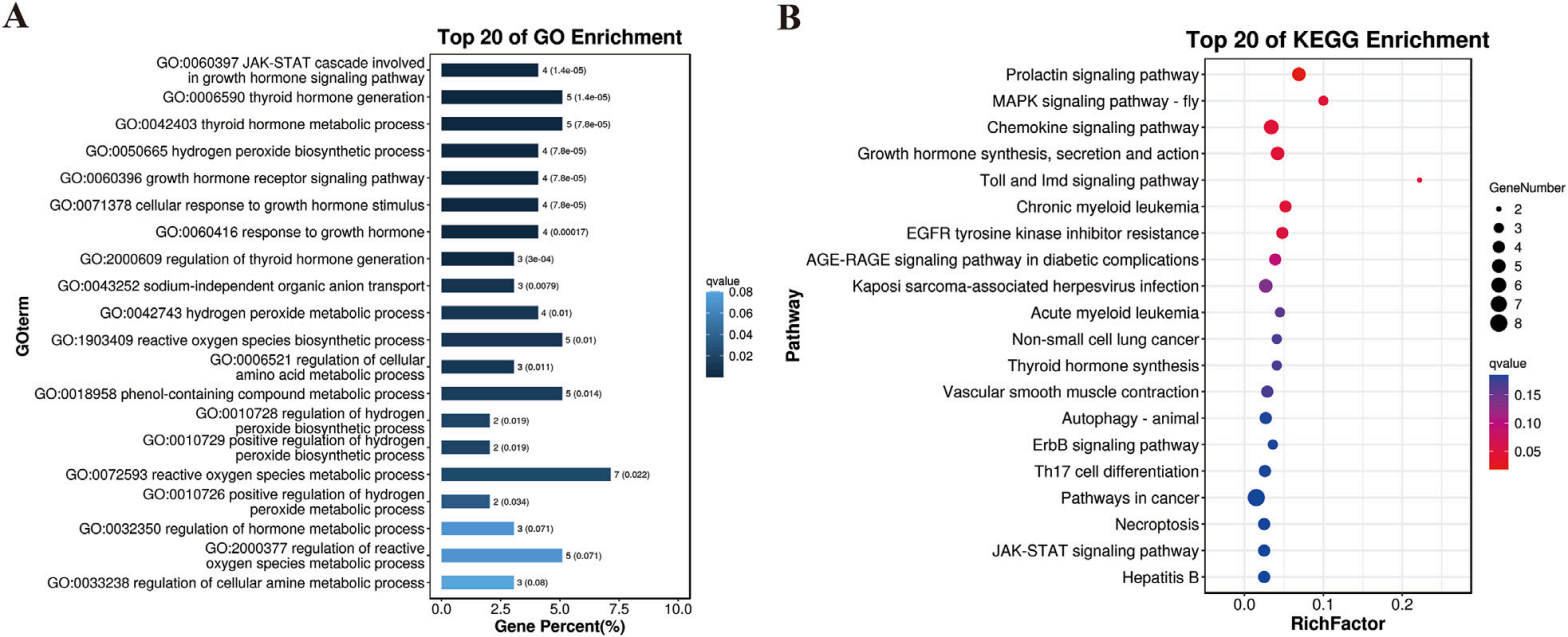
Results of the GO and KEGG analyses for the genes harbored in the selection regions. **(A)** GO terms of the identified genes. **(B)** Top 20 enrichment pathways.

Additionally, we used a combination of *F*_ST_ and π ratio analyses to identify genome-wide selective sweeps in YT compared to AWB, with both thresholds set at the top 1% level (*F*_ST_ ≥ 0.49 and π ratio ≥3.25, as shown in Figures 7A–C). We identified 321 regions associated with selective sweeps and extracted 156 candidate genes from the comparison between YT and AWB (Supplementary Table S7). Furthermore, we applied three analytical methods (*F*_ST_, π and Tajima’s D) to characterize positive selection between YT and AWB (Figure 7D). Moreover, 367 GO terms were significantly enriched (*P* < 0.05; Figure 8A; Supplementary Table S8) and associated with the JAK-STAT signaling pathway, involved in growth hormone signaling, thyroid hormones generation, hydrogen peroxide biosynthetic process and growth hormone receptor signaling pathway. In our KEGG analysis, 24 pathways were significantly enriched (*P* < 0.05; Figure 8B; Supplementary Table S9), including the Prolactin signaling pathway, MAPK signaling pathway, Chemokine signaling pathway, and Growth hormone synthesis, secretion and action.

### QTL mapping

**FIGURE 9:**
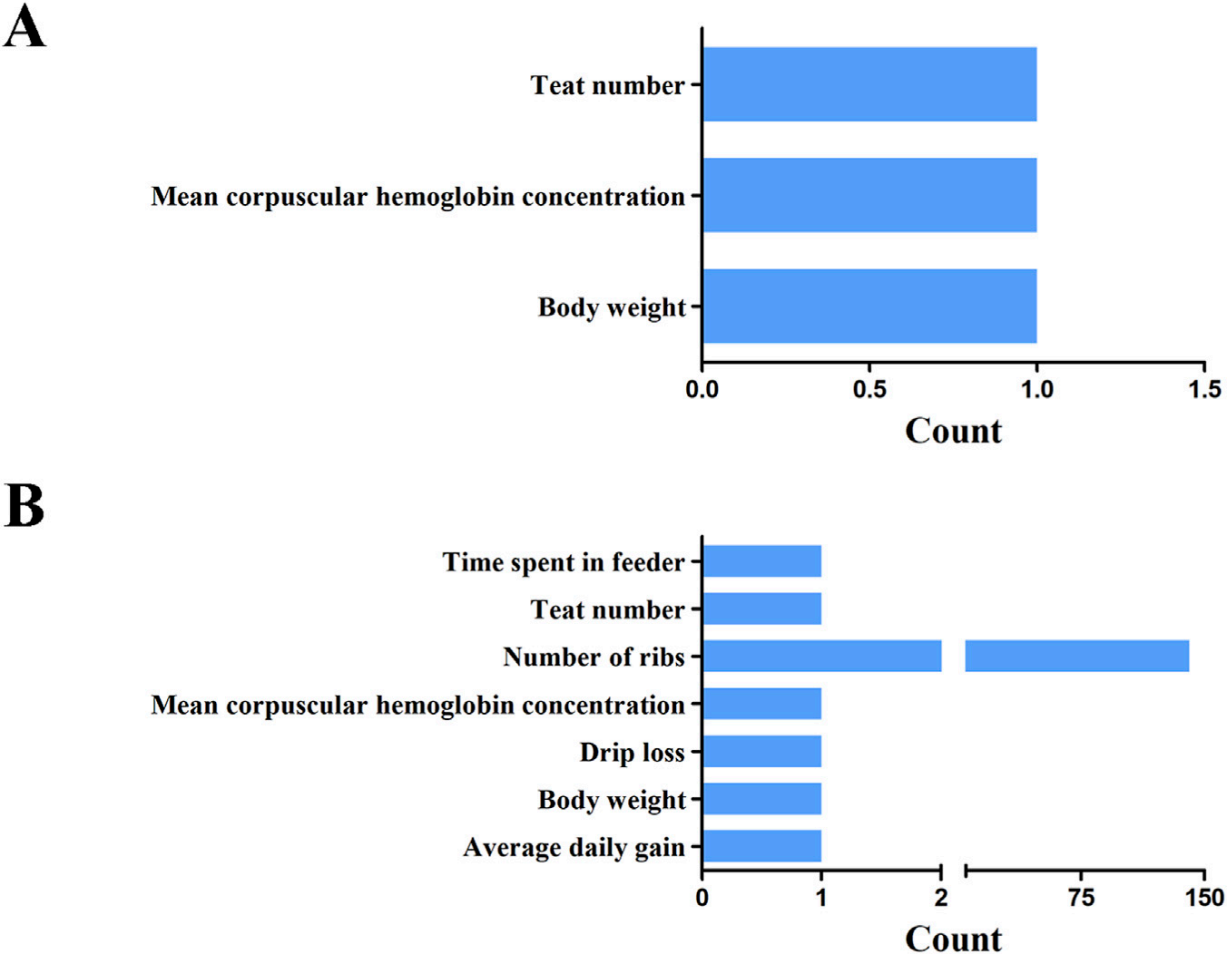
The unionset regions of selective regions, ROH islands and pig QTL of YT. Statistics on the number of QTL for different traits at the threshold level of 1% **(A)** and 5% **(B)**.

In this study, the QTL database was employed to annotate economic traits associated with overlapping regions between selection sweeps and ROH islands at various thresholds. At the 1% significance level, three overlapping regions were identified across the genome, specifically on SSC1 and SSC6. The identified QTLs were categorized into three groups: teat number, body weight, and mean corpuscular hemoglobin concentration (Figure 9A; Supplementary Table S10). Furthermore, at the top 5% threshold, 147 QTLs were identified within or overlapping these five candidate regions, associated with economically significant traits, including growth, carcass, and meat traits (Figure 9B; Supplementary Table S11). Notably, the number of QTLs associated with ribs number was significantly higher than that of other traits, accounting 95.92% of the total.

## Discussion

The pig sector holds paramount significance in global agricultural practices, with various indigenous pig populations serving as essential genetic resources. The emergence and consistent transmission of novel genetic variations are fundamental to species evolution, and identifying these variations is crucial for understanding the mechanisms driving trait diversification. Whole-genome sequencing (WGS) provides distinct advantages over high-density chips, particularly in detecting new nucleotide mutations and offering comprehensive insights into genetic markers (Tam et al., 2019). By integrating WGS data from representative pig populations worldwide, we identified 30,436,852 SNPs, achieving an alignment rate of 92.04%. This indicates that the data volume and sequencing accuracy attained in this study are sufficient for advanced genomic investigations. This study represents the most extensive dataset to date on the population genetic structure of YT, surpassing previous research in both sample size and sequencing depth.

### Genetic diversity of YT

Characterizing genetic diversity and population structure is crucial for uncovering evolutionary history, understanding environmental adaptation, conserving and utilizing germplasm resources, and exploring phylogenetic relationships. Nucleotide diversity is a widely accepted metric for assessing diversity within and among populations, serving as a quantitative measure of genetic variation (Adhikari et al., 2020). The foreign pig populations displayed reduced genetic diversity compared with the indigenous populations. Both YT (mean = 0.002501211) and MIN (mean = 0.002501117) exhibit comparable levels of nucleotide diversity, likely due to their shared genetic background (Zhang et al., 2024b). Compared to another indigenous pig population (LW) in the same area, YT has experienced a shorter period of protection and receives less breeding guidance, contributing to its relatively higher genetic diversity and lower inbreeding levels. Furthermore, the observed LD decay patterns in each population closely align with the nucleotide diversity findings. As an indigenous pig population from Shandong, YT demonstrates greater LD attenuation than other commercial and indigenous pig populations, indicating a faster decay rate. These findings suggest that YT has fewer variants, lower LD decay distance, and higher nucleotide diversity compared to other native populations, indicating distinctive genetic attributes. Although nucleotide diversity is lower in YT compared to AWB, we observed a faster LD decay pattern in YT. This pattern may be explained by demographic events, such as a population bottleneck, or by selection pressures favoring specific genetic variants, which could accelerate LD decay (Novo et al., 2022). Additionally, technical factors like sample size and SNP density might contribute to this observation. Further studies incorporating larger sample sizes and denser SNP arrays could help clarify this pattern.

PCA and NJ tree analyses indicate that YT exhibits a distinctive genetic profile among native pigs in eastern China, effectively differentiating it from both commercial pig populations and indigenous Chinese varieties. However, YT retains a minor proportion of genetic material from LW and Min pigs, consistent with historical population migration patterns (Zhang et al., 2024b). Ancestral component analysis can elucidate the extent of genetic exchange. Historically, many Chinese indigenous pig populations have been interbred with various European pig populations, leading to the indirect introgression of the Asian gene pool (Chen et al., 2020). The lineage composition of YT is complex, incorporating Western commercial pig populations (K = 8). Furthermore, Based on the individual genetic relationships, a rational breeding program can be developed to reduce inbreeding and maintain the genetic diversity of Yantai black pig.

### Runs of homozygosity characteristics analysis

Classifying ROHs based on their quantity, distribution, and specific physical lengths within the genome can provide valuable insights into the evolutionary history and extent of inbreeding within a population (Schiavo et al., 2022). Our research shows that the number of ROHs identified in YT significantly exceeds that in AWB and other pig populations (Xu et al., 2019; Wang L. et al., 2024), suggesting that reduced population size and selection pressure likely contributor to ROH formation. This variation can be partly attributed to the population’s unique genetic characteristics and the minimum length criteria for ROH, which are constrained by detection techniques. Research indicates that animals under strong selection tend to exhibit more ROHs compared to those under weaker selection pressures (Xie et al., 2019). In line with these observations, our results demonstrated significant disparities in the quantity and length of ROHs between the YT and AWB populations. Previous studies using SNP chip data have frequently identified longer ROHs (Szmatoła et al., 2020; Zhang et al., 2022). In contrast, whole-genome sequencing typically yield millions of SNPs, facilitating the detection of shorter fragments, a trend also observed in other species (An et al., 2024; Wang Z. et al., 2024). Notably, the majority of the segments (95.14%) were within the shorter length range of 0–1 Mb, aligning with previous findings in other Chinese pig populations (Zhang et al., 2023), indicating that inbreeding was more prevalent among ancient ancestors than in contemporary populations. Similarly, it is important to emphasize that SSC one exhibits the highest count of ROH, a finding also noted in research involving sheep (Ghoreishifar et al., 2019), horses (Chen et al., 2023), and chickens (Wang et al., 2023). A chromosome with low ROH coverage may suggest purifying selection, reducing the prevalence of homozygous deleterious alleles. While numerous factors influence ROH formation, their development and evolution occur randomly, largely due to recombination dynamics and gamete production randomness. Nevertheless, the genomic distribution of ROH is influenced by both individual and population-level factors during the ongoing processes of evolution and selection. For instance, pigs that have undergone inbreeding tend to exhibit a higher occurrence of long ROH.

Historically, the inbreeding coefficient has been calculated using pedigree information (*F*_PED_) (Schiavo et al., 2020). However, in practical applications, reliable and accurate ancestry data are often unavailable for these calculations, and inbreeding evaluations beyond three generations of shared ancestors are seldom performed. The advent of high-throughput genotyping technologies has enabled the use of genetic markers to provide a more precise estimate of relationships compared to pedigree data (Jiang et al., 2022; Yang et al., 2024), potentially mitigating the challenges mentioned earlier. The results for *F*_ROH_ and corresponding indices are consistent, indicating that YT has greater genetic diversity and lower inbreeding levels. Localized inbreeding within the population, due to factors such as limited mate choice or geographic isolation, can lead to homozygosity in certain genomic regions. However, introgression from external populations helps maintain overall genetic diversity. Furthermore, the effects of inbreeding are not uniformly distributed across the genome. While certain regions show increased homozygosity, loci under selection may maintain diversity. Commercial pig populations, known for their superior production capabilities, are predominantly used in intensive farming operations. Extensive artificial selection and targeted breeding have reduced their genetic diversity. The *F*_ROH_ coefficient in the YT population was lower than that in AWB but higher than in several other indigenous pig populations. Within YT pig farms, factors such as small population size, limited number of ancestors, artificial insemination, and batch management of sows hinder random mating, resulting in increased inbreeding compared to AWB pigs, resulting in an inevitable increase in inbreeding. Therefore, *F*_ROH_ may represent a more effective and precise method for assessing animal relatedness and inbreeding levels.

### Candidate gene and functional enrichment analysis

In recent years, diverse genome scanning methodologies have attracted considerable research interest, primarily because different evolutionary mechanisms generate distinct genomic signatures. Through the identification of these signatures, researchers are able to pinpoint specific genes that are vital for breeding and conservation initiatives (Hohenlohe et al., 2021). Despite the application of various statistical methods and tools to investigate the genomic patterns of different livestock and poultry species, a comprehensive literature that details the entire YT genome is still lacking. Following wild boars domestication, domestic pigs have undergone intense selective pressure to cultivate preferred phenotypes. YT represents a valuable asset for advancing animal husbandry, given its remarkable attributes, particularly its exceptional reproductive performance, high-quality meat, and resistance to rough feeding conditions. By comparing YT genomic data with that of wild boars, it is possible to identify the genomic regions and relevant genes involved in domestication and breed selection.

To enhance assay efficiency while minimizing false positives, three targeted detection methods for signatures were applied to YT. The distribution of ROH across the genome varies by population, with most ROHs concentrated in specific regions forming clusters known as ROH islands. Unlike other genomic regions, ROH islands exhibit reduced genetic diversity and increased homozygosity. As a result, these ROH islands are subject to selective pressures, particularly when candidate genes within these regions undergo adaptive selection. Consequently, the candidate gene identified in this research based on ROH islands may play a crucial role in tracking genetic changes resulting from selection in the YT population. Numerous genes are present in the ROH islands of both YT and AWB genomes, with GO and KEGG analyses indicating that genes in YT ROH islands are primarily enriched in pathways related to Glycan biosynthesis and metabolism, Digestive system, and Lipid metabolism. Compared to commercial pig populations, Chinese indigenous pigs generally exhibit a greater propensity for fat deposition. Therefore, we propose that degradation of glycosaminoglycans may serve as a mechanism to mitigate lipidosis progression. YT pigs are provided with high-fiber feed options such as sweet potato leaves and protein-rich mulberry. Increasing dietary fiber content enhance pancreatic juice volume, potentially due to the non-cellulosic components of dietary fiber.

Wild boars and other large mammals were important prey for early hunter-gatherers. Domesticated pigs exhibit significant changes in physiology, morphology, and behavior compared to their wild ancestors. Over their long history of evolution and breeding, pigs have undergone both natural and artificial selection to fulfill various human needs worldwide. The genomic regions and associated genes involved in pig domestication and breed selection can be identified by comparing the genomic information of domestic pigs and wild boars. The genomic regions and related genes involved in domestic pig domestication and breed selection can be obtained by comparing the genomic genetic information of domestic swine and wild boars. *F*_ST_ and π ratio analysis are commonly used to detect selection signals in livestock (Qin et al., 2020; Yu et al., 2024). In Zhu’s research, *F*_ST_ and π ratio analyses were integrated to delineate the genomic selection region of Zhaotong pigs, uncovering selected genes relevant to fat deposition, reproduction, and growth (Zhu et al., 2024). In this study, *F*_ST_ analyses were used to identify selective sweeps and pinpoint the targets of positive selection in the genomes of YT and AWB. Functional enrichment analyses indicated that these selected genes may significantly influence growth, reproduction, and immune responses. Chinese indigenous pig populations exhibit common biological traits, such as enhanced litter size and unique estrus behaviors. Compared to commercial pig populations, most Chinese indigenous populations reach puberty earlier, exhibit longer behavioral estrus periods, and have slightly shorter estrus cycles, with female YT gilts maturing around 5 months of age. Reproductive endocrinological and genetic factors play a crucial role in regulating puberty. Transcriptomic analyses comparing Wanyue Black gilts and Yorkshire gilts revealed that differentially expressed genes (DEGs) are involved in various signaling pathways related to hormone production and puberty, including Steroid hormone biosynthesis, Estrogen signaling pathway, and Prolactin signaling pathway (Zhang W. et al., 2024). Furthermore, prolactin, a multifunctional protein hormone, is involved in appetite regulation, immune modulation, growth regulation, and the stress response. Gao et al. indicated that certain upregulated chemokines can attract more immune cells for pathogen defense, with Duroc pigs exhibiting enhanced chemotactic immune cell capabilities (Gao et al., 2018). The Toll and Imd signaling pathways are well-established immune signaling pathways that facilitate the transcription and production of most antimicrobial peptides (AMPs) and other active compounds.

Pavlidis et al. emphasized that genome scans analyses for selection are constrained by the absence of a prior hypothesis, leading to a vast array of possible interpretations (Pavlidis et al., 2012). Furthermore, gene functional enrichment analysis typically prioritizes biological aspects, complicating the elucidation of inherent gene-trait connections. QTL mapping effectively accounts for genetic interactions among molecular markers and the impact of fixed and random effects on phenotypes. Therefore, integrating QTL scans with selection mapping could effectively narrow down potential hypotheses to those specifically aligned with the trait in question. In this study, the pig QTL database was employed to annotate economic traits associated with overlapping regions between sweep selection areas and ROH islands. The findings indicated that most overlapping regions corresponded to QTL associated with growth (ID 153574 for *WDR27* and *THBS2*, and ID 171471 for *RBFOX3*), reproduction (ID 295053 for *WDR27* and *THBS2*), carcass (ID 273550 for *PTGR2*), immune response (ID 27351 for *CALM3*, *PTGIR*, *GNG8*, *DACT3*, and *PRKD2*), and meat quality (ID 21947 for *WDR27* and *THBS2*). Previous studies indicate that YT exhibits a variety of resilient traits, including superior growth performance, forage resistance, and robust disease resistance (Huang et al., 2016). Consequently, it has become a reputable source of healthy and safe meat, significantly contributing to the economic advancement of the pig industry in China. The functional annotation of potential genes and the overlap of QTL are both associated with YT characteristics. In the GWAS focused on meat quality traits in Yorkshire pigs, the SNP locus MARC0110724, located within the intron of *WDR27*, is associated with drip loss and water loss rates (Dong, 2015). Among identified fertility candidate genes in cattle, *WDR27* also features the highest confidence variants mapped to it. Furthermore, Raven et al. indicated a correlation between the *WDR27* candidate gene and cattle survival rates (Raven et al., 2014). Following the GLS analysis of EPISNP2, the two SNPs located upstream of *PTGR2* exhibited the most significant additive effects on the number of teats in Duroc pigs, achieving genome-wide significance (Tan et al., 2017). Similarly, a combined analysis of GWAS and LD results revealed a significant correlation between the *PTGR2* gene and total teat number in both Danish (SSC7 91.19–97.80 Mb) and American (SSC7 97.08–98.09 Mb) Yorkshire populations (Guo, 2019). The *CALM3* gene, part of the calmodulin gene family, is essential for mediating a range of processes, including inflammation, immune responses, apoptosis, and metabolic pathways (Shao et al., 2025). Additionally, *PTGIR* is involved in regulating inflammatory responses and is expressed in a variety of immune cells, including T cells, B cells, and macrophages. Modulating *PTGIR* expression may affect the equilibrium between pro-inflammatory and anti-inflammatory responses, thereby influencing overall immune cells activity within the airways (Na et al., 2021). *PRKD2*, a member of the serine-threonine kinase family, is crucial for the survival, proliferation, migration, and angiogenesis of tumor cells (Zhang et al., 2025). *RBFOX3*, also known as NeuN, is a recognized neural nuclear antigen capable of binding to DNA *in vitro*. It serves as an effective marker for mature neurons in both central and peripheral nervous systems and plays a crucial role in neural cells differentiation (Duan et al., 2016). It is worth noting that loss of NeuN immunoreactivity in spinal cord neurons of rats during the aging process (Portiansky, et al., 2006). Therefore, *WDR27* and *RBFOX3* may be important genes affecting the growth and immune response of Yantai Black pig.

## Conclusion

This study revealed that Yantai Black pig had a unique population structure, relative high genetic diversity, and obvious genetic differentiation within the population from the whole-genome sequence. Selected regions contain a large number of genes associated with immunity, reproduction, growth and development, highlighting the need for their scientific and effective protection as valuable germplasm resources. The breed conservation program remains to be further optimized so as to ensure adequate genetic diversity and avoid inbreeding depression. Our research findings enhance the understanding of the inherited mechanisms underlying economical traits and offer guidance for future genetic improvement and breeding conservation planning of Yantai Black pig.

## Data Availability

The data presented in the study are deposited in the NCBI repository (https://www.ncbi.nlm.nih.gov/sra/), project number PRJNA1258694.
