# Supplementary material for: Genomic analysis reveals genetic diversity and selection signatures of the Yantai Black pig during domestication and breeding
Source: Front Genet. 2025 Nov 25;16:1730668. doi: 10.3389/fgene.2025.1730668 (PMC12685829; doi:10.3389/fgene.2025.1730668)
Supplement: Supplementary file 1 [file DataSheet1.docx]

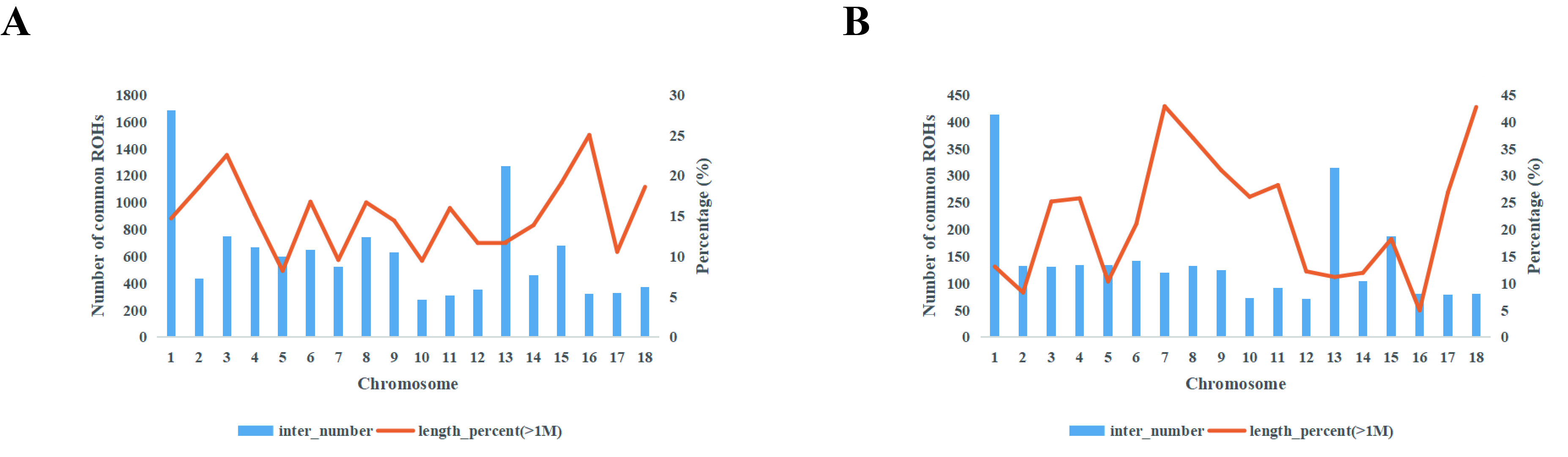


**Supplementary Figure S1.** Number of common runs of homozygosity (ROH) and >1 Mb ROH length rate (red line) in the chromosomes of YT (A) and AWB (B).

**
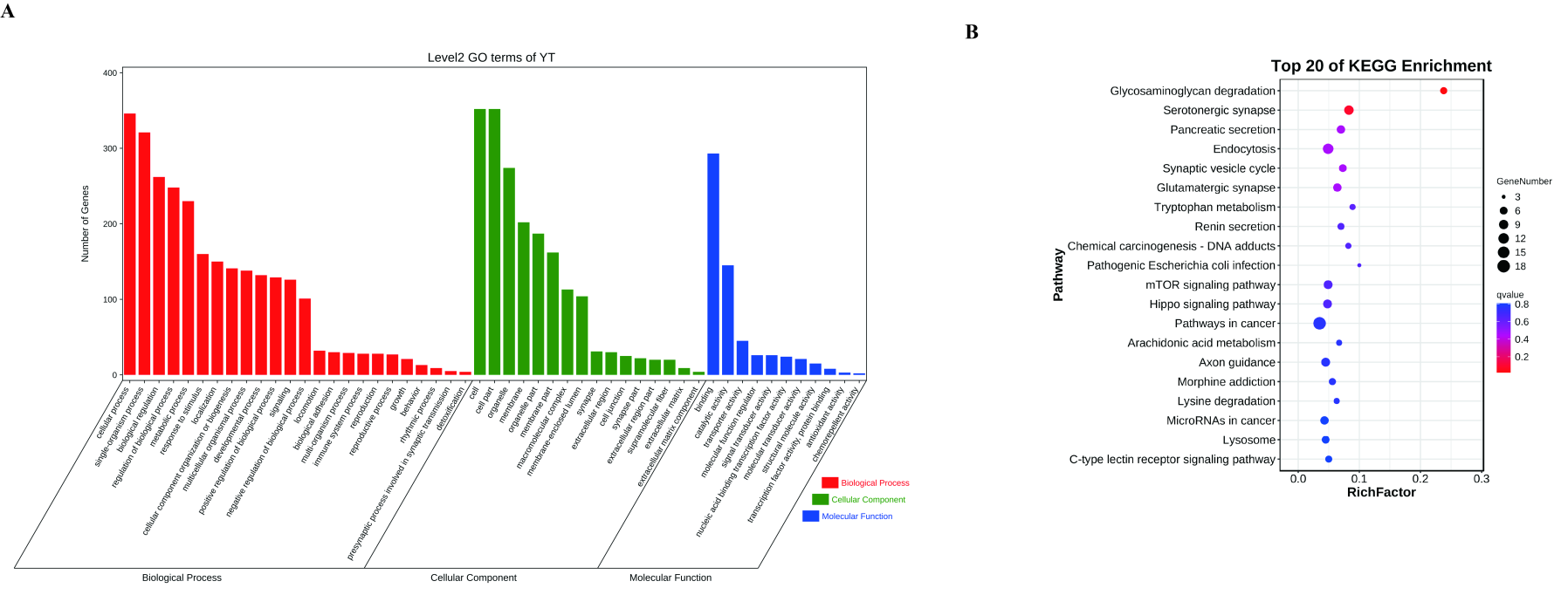
**

**Supplementary Figure S2.** GO (A) and KEGG (B) enrichment analysis of genes in run of homozygosity (ROH) islands of YT.
